# Quantitative Imaging of Regional Cerebral Protein Synthesis in Clinical Alzheimer's Disease by [^11^C]Leucine PET

**DOI:** 10.1007/s11307-024-01965-3

**Published:** 2024-11-20

**Authors:** Karl Herholz, Adam McMahon, Jennifer C. Thompson, Matthew Jones, Herve Boutin, Jamil Gregory, Christine A. Parker, Rainer Hinz

**Affiliations:** 1 Faculty of Biology, Medicine and Health, School of Biological Sciences, Division of Neuroscience, University of Manchester, Manchester, M13 9PL UK; 2 Faculty of Biology, Medicine and Health, School of Health Sciences, Division of Informatics, Imaging and Data Sciences, University of Manchester, Manchester, M13 9PL UK; 3 Manchester Centre for Clinical Neurosciences, Salford, M6 8HD UK; 4 UMR 1253, iBrain, Université de Tours, Inserm, UFR de Médecine, 10 Bd Tonnellé, 37032 Tours Cedex 01, France; 5 GlaxoSmithKline, Gunnels Wood Road, Stevenage, SG1 2NY UK

**Keywords:** Alzheimer’s disease, Positron emission tomography, Cerebral protein synthesis, Amino acids

## Abstract

**Purpose:**

Protein synthesis is essential to maintain integrity and function of the human brain, and protein synthesis is associated specifically with the formation of long-term memory. Experimental and clinical observations indicate that this process is disturbed in Alzheimer’s dementia and other neurodegenerative diseases. *In-vivo* investigation with positron emission tomography (PET) using [^11^C]leucine provides a unique possibility to measure regional cerebral protein synthesis (rCPS) rates in human brain and to determine whether it is altered in Alzheimer’s disease (AD), and thus may provide a target for future therapeutic interventions.

**Procedures:**

In this first human study, we measured rCPS by [^11^C]leucine PET in four patients with AD (age 57–73 years) and compared the results with six healthy controls (three of whom were age matched and the other three were young controls). Quantification of rCPS also required measurement of amino acid (AA) levels and of free and protein-bound [^11^C]leucine in plasma during the 90 min PET scans conducted following at least six hours of fasting.

**Results:**

Rates of rCPS measured in absolute units of nmol/g/min ranged between 1.81 and 2.53 in AD patients, 2.10 and 2.54 in matched controls, and 2.21 to 2.35 in the young controls. Mean and median values did not show significant differences between the groups. Rates of rCPS also depended upon whether corrections for plasma AA levels were included in the calculations. When considering regional values relative to the corpus callosum as a reference region, there was a tendency towards impairment of rCPS in patients, which was most prominent in the parietal cortex, but did not reach significance. Similar findings were observed with normalisation of rCPS to global cortical mean.

**Conclusions:**

In summary, this first human study assessing regional protein synthesis with [^11^C]leucine in AD has demonstrated where the sources of variance in measurements of cerebral protein synthesis may arise, along with the potential magnitude of this variance. This study also indicates that there is a tendency towards impairment of rCPS in patients with Alzheimer’s disease, which requires further investigation including possible partial volume effects due to atrophy.

**Supplementary Information:**

The online version contains supplementary material available at 10.1007/s11307-024-01965-3.

## Introduction

Protein synthesis is a core function of all body cells that is essential to viability. Imaging of protein synthesis rates therefore is of considerable interest in clinical research for many diseases, including Alzheimer’s disease (AD). This is possible with positron emission tomography (PET), which otherwise is widely used for imaging of glucose metabolism and deposition of pathological proteins, especially beta-amyloid and tau, in AD [1]. There are also radiotracers enabling research PET imaging of other physiological measures, such as blood flow, energy metabolism [2], neuroinflammation [3], and protein synthesis [4] in AD.

In the brain, protein synthesis is specifically required for the formation of long-term memory through long-term potentiation and long-lasting reduction in post burst after hyperpolarization [5]. Conversely, protein synthesis rates are reduced substantially in cells undergoing apoptosis and after exposure to cellular stress [6]. While the reduction of protein synthesis rates as part of the heat shock response [7] is a highly conserved acute protective reaction, prolonged reduction of protein synthesis is associated with delayed cell death [8]. Even transient ischemia may result in long-term inhibition of translation [9, 10] and subsequent cell death.

Protein synthesis rates depend on transcription and translation. Changes in both are associated with synaptic plasticity [11] and protein synthesis is essential for neurotrophin-dependent neuroplasticity of dendritic spines [12]. Studies in rats have demonstrated that cerebral protein synthesis rates are reduced already at mid-age compared to young adults [13, 14]. Many inherited neurological diseases are associated with quantitative alteration of protein translation [15]. In neurodegenerative disease, e.g. in prion disease and Alzheimer’s disease (AD), regional cerebral protein synthesis (rCPS) may be suppressed by the unfolded protein response (UPR) and increased activity of the protein kinase RNA-like endoplasmic reticulum kinase (PERK) pathway [16, 17]. Reductions of several genes encoding ribosomal proteins and eukaryotic elongation factors 1A and 2 were observed in AD [18].

The main pathological hallmarks of AD, β-amyloid plaques, neurofibrillary tangles (NFT), neuronal and synaptic loss and chronic neuroinflammation are associated with changes in mRNA translation, oxidative stress, endoplasmic reticulum (ER) and mitochondrial dysfunction [19, 20]. These pathologies promote the activation of the UPR pathway which has been found in post-mortem brains of AD patients and also in animal models of AD [17, 21, 22]. It attenuates protein synthesis which, if prolonged, may cause cellular dysfunction and death [23]. As a result, the UPR pathway in AD could represent a potential therapeutic target of interest [24]. Changes of amino acid (AA) profiles in plasma and cerebrospinal fluid that may indicate alterations of protein synthesis have been observed previously in patients with AD [25, 26].

The PET imaging technique for measuring rCPS rates in humans using [^11^C]leucine has been developed and thoroughly validated in children and young healthy subjects [27–33]. Ninety percent of radiolabelled leucine entering the brain from blood plasma was demonstrated to be incorporated into proteins [34]. [^11^C]Methionine has been used previously in AD as an alternative means to measure protein synthesis with PET [35, 36], however, this tracer partially enters into complex metabolic pathways that are unrelated to protein synthesis. This complicates kinetic modelling of [^11^C]methionine and accurate quantification of PSR with this tracer.

This manuscript describes a proof of principle clinical study measuring protein synthesis dysfunction in AD using [^11^C]leucine PET. It required implementation and optimisation of a complex non-invasive method for application in the AD patient group, with the ultimate goal of enabling this technique to be used for the assessment of new therapeutic strategies in future clinical trials. As noted, studies to date have indicated the utility of [^11^C]leucine PET to quantify rCPS in healthy subjects. [^11^C]Leucine PET has recently also been used in a transgenic rat model of AD [37] but, to our knowledge, has not been applied to patients with AD, making this the first report presenting data from a clinical AD cohort.

The specific aims of this study were to demonstrate the feasibility of measuring rCPS in AD patients with [^11^C]leucine PET and to compare these data and their variance with age-matched and young healthy subjects.

## Participants

Ten participants were investigated with PET in this study. These included four patients with mild to moderate AD, meeting the clinical criteria of the National Institute on Aging and the Alzheimer’s Association workgroup for probable AD [38]. They had significant memory impairment, Mini Mental State Examination (MMSE) scores 10 to 24. Inclusion criteria also required a positive amyloid PET scan. Three elderly healthy subjects were included as controls, and also three young healthy subjects to account for a potential age effect and to compare results with previously published studies in young healthy subjects. Comprehensive exclusion criteria comprised active concurrent metabolic, neurologic or psychiatric diseases. Eligibility was examined initially by telephone interview and subsequently by clinical examination and blood tests. Neuropsychological examination of AD patients and elderly controls included MMSE, CDR, and ADAS-cog (Table 1).

**Table 1:** Participant demographics (median and range or frequency)

Group	Age (years)	Age range	Sex	Age at onset	CDR	MMSE	MMSE range	ADAScog	ADAScog range
Young controls	24	23–24	1 M/2F	N/A	N/A	N/A	N/A	N/A	N/A
Old controls	58	57–58	2 M/1F	N/A	0	30	27–30	3.66	2–4
Patients	66	57–73	2 M/2F	58 (55–66)	1	15	14–23	25.6	16–43

Ethical study approval was obtained from the North West—Greater Manchester South Research Ethics Committee (Ref. 16/NW/0705), and permission to administer radioisotopes was obtained from the Administration of Radioactive Substances Advisory Committee (ARSAC) of the UK (Ref. 16/NW/0705). The study is registered in clinical trials.gov (NCT05491902).

## Methods

The following is a brief description of methods. A detailed description is provided in the digital supplement.

L-[1-^11^C]Leucine (termed [^11^C]leucine throughout the rest of the manuscript for brevity) was synthesized in a hot cell on a GE Tracerlab using a modified Bucherer-Strecker AA synthesis [39]. HPLC gave an enantiomeric purity > 96%.

All PET scans were performed on a High Resolution Research Tomograph (HRRT, CTI/Siemens Molecular Imaging, Knoxville, Tennessee). After i.v. bolus injection of 641 MBq [^11^C]leucine (range 520 to 783 MBq) with radiochemical purity of 98.0% to 99.4% and specific activity of 16.4 GBq · μmol^−1^ (SD 8.6 GBq · μmol^−1^) a 90 min dynamic scan was recorded in list-mode. The 90 min dynamic scan was binned into 43 frames which were reconstructed with the iterative ordinary Poisson ordered-subset expectation maximization (OP OSEM) 3-D algorithm, incorporating normalization and corrections for random coincidences, scattered radiation and attenuation [40]. After reconstruction, images were regularized with a 3-D Gaussian filter of 2 mm full-width at half-maximum to reduce image noise.

Arterial whole-blood activity was monitored continuously for the first 15 min of the scan with a bismuth germanate coincidence detector. During the 90 min scan, a total of 11 discrete arterial blood samples were taken and the activity concentration of the whole blood and plasma were measured. Plasma samples were also analysed for the parent fraction of [^11^C]leucine unbound to plasma protein. However, due to technical problems described in the supplement the parent fraction could not be determined reliably. Therefore, a population mean curve for the fraction of unbound [^11^C]leucine in plasma as published by Sundaram et al*.* [33] was used convert measured total plasma activity to parent plasma activity (Supplementary Fig. 1).

For each participant, brain magnetic resonance images (MRI) were acquired to provide structural volumetric T1 images for subsequent segmentation and atlas-based generation of volumes of interest on a 1.5 Tesla Philips Achieva scanner. PET and MRI scans were co-registered. A probabilistic atlas [41] was used to generate bilateral VOIs (13 grey matter, 1 white matter) adapted from the report by Bishu et al*.* [30] for the kinetic analysis of [^11^C]leucine.

An irreversible two-tissue compartment model as specified by Sundaram et al*.* [33] and the fractional blood volume as free model parameter was used for the calculation of the following outcome parameters:

1. The unidirectional uptake rate constant of plasma leucine into tissue, K_cplx_,
2. The fraction of intracellular leucine originating from plasma, λ,
3. The protein synthesis rate (rCPS), calculated by multiplication of K_cplx_ with the activity of unlabeled leucine in plasma divided through λ.

Older healthy subjects and AD patients also underwent an amyloid PET scan with 150 MBq [^18^F]flutemetamol (GE Healthcare, Amersham, UK) recorded 90 to 110 min after tracer and evaluated visually according to standard clinical criteria [42].

Kruskal–Wallis tests were used to examine differences of outcome parameters among groups (young controls vs old controls vs patients) in individual regions. Differential effects of groups on regional PSR were analysed using mixed effect models and Chi-square tests. Global cerebral grey matter values were calculated as the average across supratentorial grey matter regions (all regions except cerebellum and corpus callosum) weighted by region size.

## Results

### Clinical Characteristics and Plasma Amino Acids

In total 18 participants were recruited and underwent full screening. Ten of them fulfilled eligibility criteria and completed MR and PET scanning. As detailed in Table 1, these included 3 young healthy subjects, 3 older healthy subjects and 4 AD patients. There were no drop-outs or serious adverse events.

Older healthy subjects all showed negative amyloid scans, while all AD patients demonstrated positive amyloid scans upon standard visual evaluation.

Analysis of unlabelled AAs showed results similar to previous reports in the literature [25, 43] (Tables 2 and 3). There was a tendency towards higher values in male than in female participants, and young healthy subjects tended to have lower levels than older healthy subjects. However, none of these differences reached significance and, due to the small number of subjects and the imbalance of sexes in the age groups, cannot be separated from each other.

**Table 2:** Plasma levels of unlabelled amino acids (sum of 9 LNAA, µmol/L)

Group	median	range	Mean	S.D	S.E
All	1208.93	1079–1461	1222.37	122.77	38.82
Female	1172.14	1079–1234	1156.31	62.05	27.75
Male	1299.56	1092–1461	1288.44	138.39	61.89
Young controls	1109.09	1092–1186	1129.52	50.40	29.10
Old controls	1230.93	1079–1461	1257.33	192.61	111.20
AD patients	1266.83	1172–1357	1265.80	80.22	40.11

**Table 3:** Plasma levels of unlabelled leucine (µmol/L)

Group	median	range	Mean	S.D	S.E
All	112.38	90–131	110.20	13.31	4.21
Female	111.28	90–131	109.98	16.43	7.35
Male	113.47	91–118	110.42	11.34	5.07
Young controls	98.03	91–131	106.92	21.67	12.51
Old controls	111.28	110–118	113.29	4.23	2.44
AD patients	116.11	90–118	110.34	13.66	6.83

### Quantitative PET Imaging Results

**Fig. 1:**
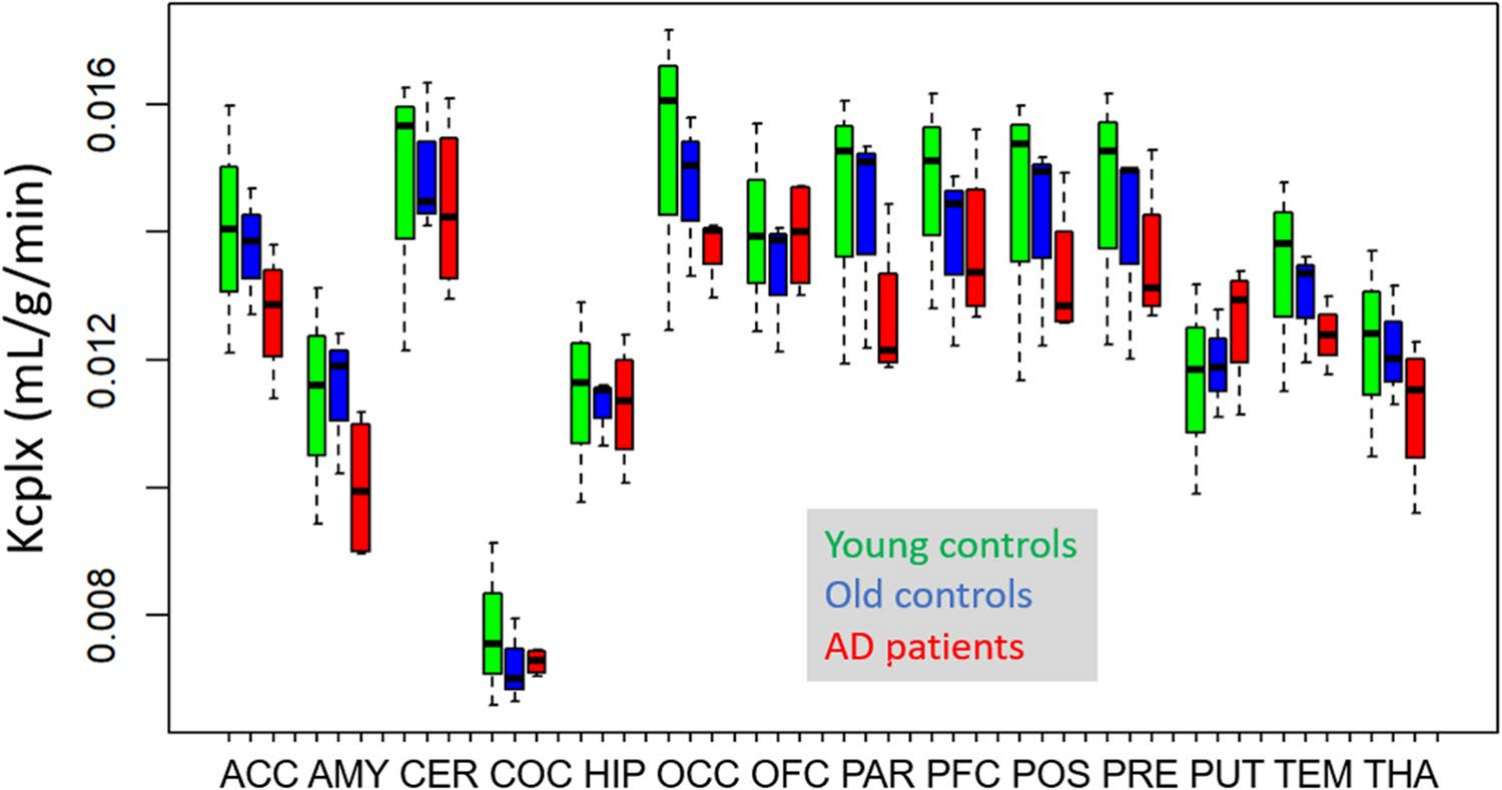
Unidirectional uptake rate of [11C]leucine (box plot). Abbreviations used for regions in this and subsequent figures are: ACC, anterior cingulate cortex; AMY, amygdala; CER, cerebellum; COC, corpus callosum; HIP, hippocampus; OCC, occipital cortex; OFC, orbitofrontal cortex; PAR, parietal cortex; PFC, prefrontal cortex; POS, postcentral gyrus; PRE, precentral gyrus; PUT, putamen; TEM, temporal cortex; THA, thalamus

As described in the methods section, K_cplx_ is a macroparameter describing cellular uptake and metabolism of [^11^C]leucine, not including corrections for unlabelled plasma AAs. As shown in Fig. 1, there was a tendency towards lower values of K_cplx_ in most but not all brain regions in AD patients compared with healthy subjects. Statistically, differences between participant groups did not reach significance. As expected, lowest values in all groups were recorded in the corpus callosum, representing white matter, with values determined as 0.0078 ± 0.0013 (mL/g/min, mean ± S.D.) in young healthy subjects; 0.0072 ± 0.0007 in older healthy subjects and 0.0073 ± 0.0002 in AD patients. Average values across cortical regions and putamen (weighted by region size) were 0.0143 ± 0.0019 in young healthy subjects, 0.0138 ± 0.0013 in older healthy subjects, and 0.0132 ± 0.0004 in AD patients (with no significant difference observed between groups). The most prominent difference was seen in the parietal region with 0.0144 ± 0.0022 in young healthy subjects, 0.0142 ± 0.0018 in older healthy subjects, and 0.0127 ± 0.0012 in AD patients, but still not reaching significance (p = 0.328, Kruskal–Wallis Test). In the mixed model analysis, there was no significant main effect between groups (p = 0.671), but a significant interaction of groups and regions (p = 0.0039).

Intracellular AAs originate from plasma and partially also from intracellular protein degradation. An estimate of the proportion originating from plasma, λ, is calculated from kinetic tracer uptake rates. It did not show differences between groups but clear regional differences without significant interaction with groups (Supplementary Fig. 2). The average value of λ across all regions and groups was 0.6770 ± 0.0314 (mean ± S.D.), with lowest values in amygdala (0.6175 ± 0.0398) and hippocampus (0.6218 ± 0.0405) and highest values in putamen (0.7424 ± 0.0299).

**Fig. 2:**
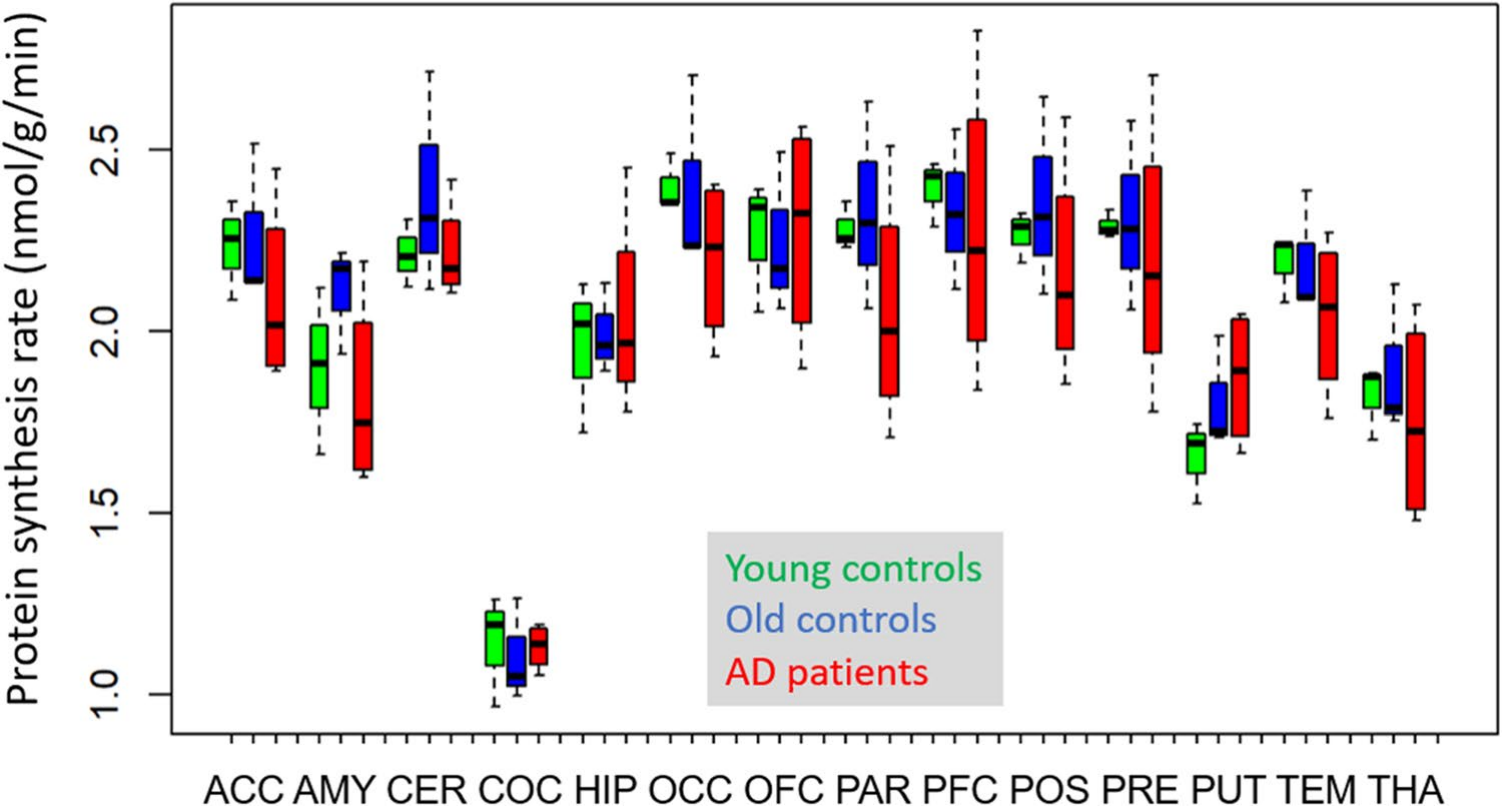
rCPS without correction for LNAA plasma levels (box plot)

Regional cerebral rates of protein synthesis (rCPS, nmol/g/min) based on PET kinetic data (K_cplx_) and individual unlabelled leucine plasma levels without correction for plasma LNAA levels showed regional differences with lowest values, as expected, in corpus callosum and consistently higher values in all grey matter regions (Fig. 2). As shown in Table 4, there were no significant differences of cortical and putamen average values between groups, and there was no significant main effect of groups or interaction with regions in the mixed model analysis.

**Table 4:** Cerebral protein synthesis rates (rCPS, nmol/g/min)

Group	Median	range	Mean	S.D	S.E
All	2.23	1.81–2.54	2.23	0.22	0.07
Young controls	2.31	2.21–2.35	2.29	0.07	0.04
Old controls	2.23	2.10–2.54	2.29	0.23	0.13
AD patients	2.13	1.81–2.53	2.15	0.31	0.15

**Fig. 3:**
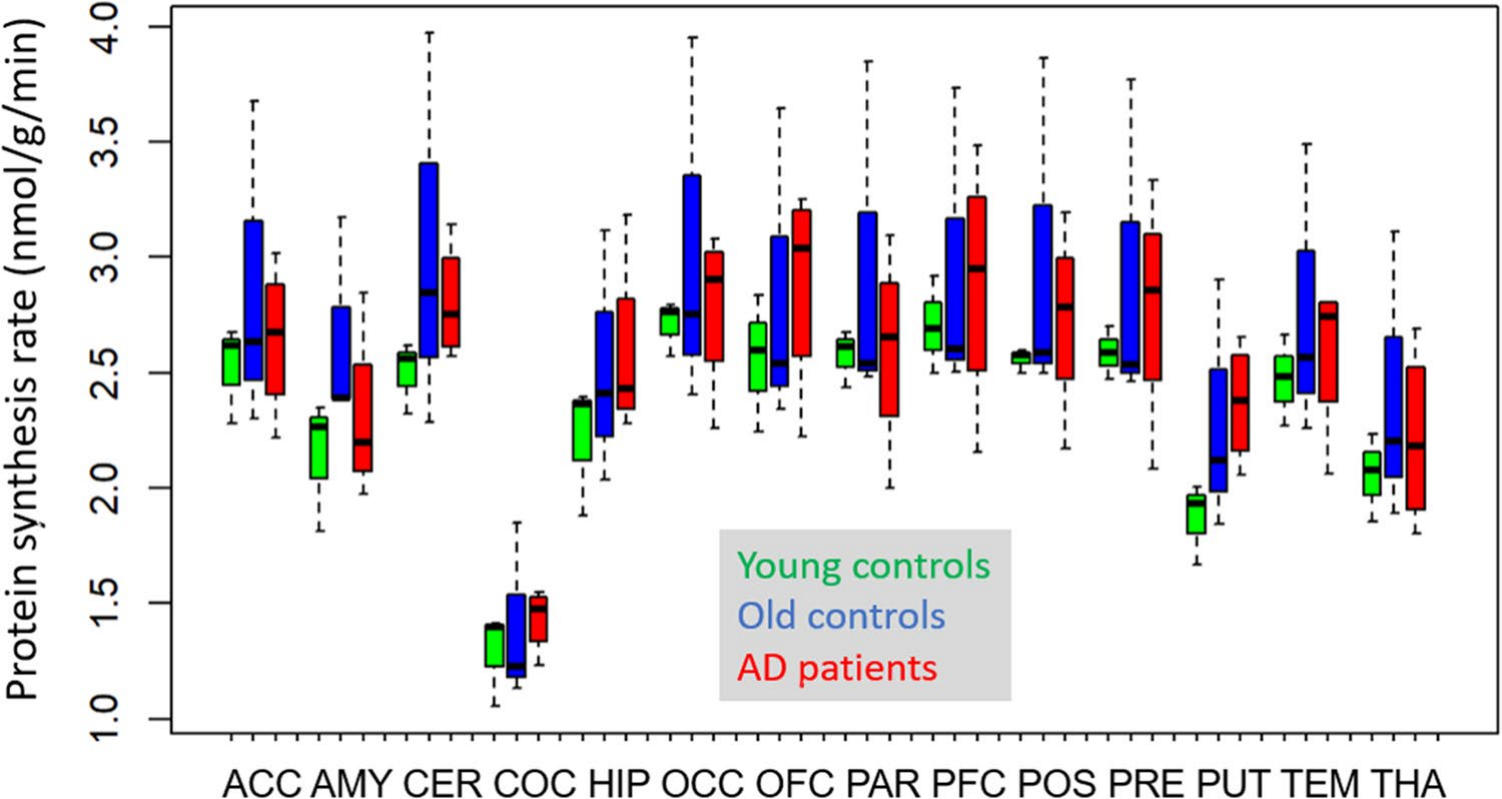
rCPS with correction for plasma LNAA levels (box plot)

Results remained essentially the same without significant differences between groups when normalising rCPS to a LNAA level of 1000 µM, as suggested by Sundaram et al. [33]. Normalised metabolic rates were generally about 20% higher than without correction (Table 5). Data variance was smaller in the AD group but larger in old controls than without normalisation (Fig. 3).

**Table 5:** Cerebral protein synthesis rates normalised to LNAA plasma levels (nmol/g/min)

Group	Median	range	Mean	S.D	S.E
All	2.67	2.12–3.72	2.73	0.44	0.14
Young controls	2.61	2.41–2.47	2.59	0.17	0.10
Old controls	2.58	2.40–3.72	2.90	0.71	0.41
AD patients	2.82	2.12–3.12	2.72	0.43	0.22

**Fig. 4:**
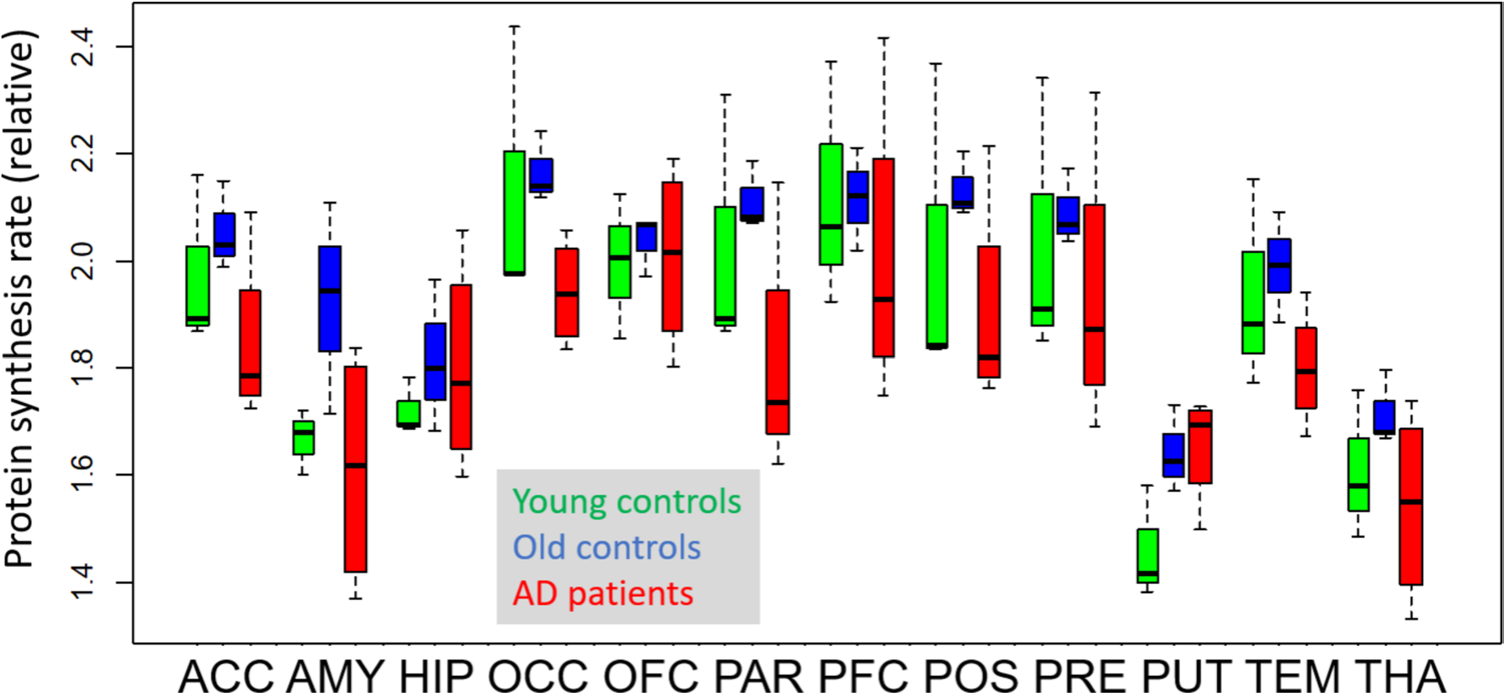
Regional rCPS relative to corpus callosum (box plot)

To eliminate the effect of factors affecting rCPS in the entire brain, including the large variation of unlabelled AA plasma levels, we calculated rCPS values relative to corpus callosum as a reference. There was a tendency towards lower values in most regions in patients, especially when compared to older controls (Fig. 4). Average grey matter values (mean ± S.D.) were 2.03 ± 0.23 nmol/g/min in younger healthy controls, 2.08 ± 0.06 in older healthy controls, and 1.89 ± 0.19 in AD patients. When focussing on parietal cortex as a particularly vulnerable region in AD, we observed relative rCPS values 2.02 ± 0.25 in younger healthy controls, 2.11 ± 0.07 in older healthy controls, and 1.81 ± 0.23 in AD patients (p = 0.215, Kruskal–Wallis test).

## Discussion

Calculation of rCPS involves complex modelling and measurement of unlabelled AA levels in plasma. In accordance with published data [25], we observed considerable variation of LNAA plasma levels among participants but no major differences of mean values between patients and healthy controls. Variation in LNAA plasma levels in older participants, and especially in older healthy subjects, tended to be larger than in younger healthy subjects. Plasma levels of LNAA including unlabelled leucine were higher in all groups than reported by Sundaram et al. [33]. However, in their study glucose loading before scanning was used to reduce competing LNAA levels [44]. We chose not to implement glucose loading as there is insufficient information whether this might cause a systemic metabolic bias between our study groups. This was in line with Bishu et al. [30], and rCPS values in young controls in their study were similar to those observed in the present study. Only when correcting rCPS values for competing LNAA (Table 5, Fig. 3), as suggested by Sundaram et al. [33], we found substantial higher values than reported in previous studies [30, 33] as a consequence of the higher LNAA values.

The dependency of measured cerebral rCPS on model configuration and variance of plasma AA levels currently limits interpretation of their linkage with neuronal function and requires further research. Plasma leucine levels influence protein synthesis rates in muscle and fat tissue via the mTOR pathway and, according to recent experimental findings [45], also in the brain. Thus, the high rCPS rates we found in correspondence to relatively high LNAA values, especially when using the rates with correction for plasma LNAA levels, most likely represent actual synthesis rates. Substantial variation of cerebral metabolic rates associated with the variation of systemic parameters has also been observed with other quantitative PET measurements, e.g. with FDG for measuring cerebral glucose metabolism [46], and therefore brain reference regions are commonly used for normalisation. We chose a large white matter structure, the corpus callosum, as reference region because synaptic density is much smaller in white than in grey matter. Corpus callosum has successfully been validated as a reference structure for [^11^C]-PIB [47], and is has also been used with other tracers for which no suitable grey matter structure could be identified as reference [48]. Mean normalised rCPS values were substantially lower (by 9%) in AD patients than in elderly controls, especially in the parietal cortex (by 14%). This was not statistically significant in our very small sample, and group sizes larger than 12 subjects would likely be required for proper statistical power.

**Fig. 5:**
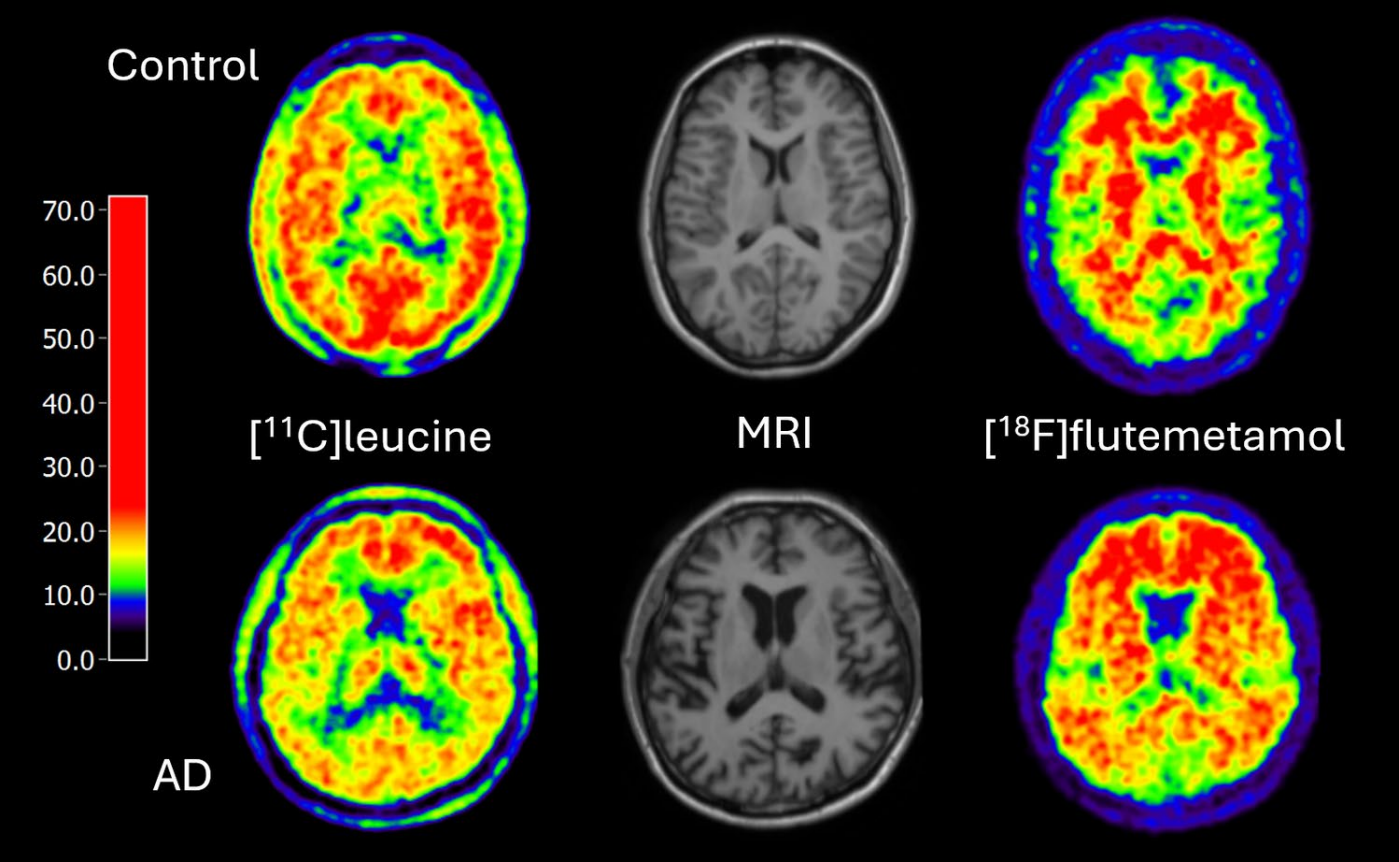
Comparison of selected illustrative scans from a control subject and an AD patient, both 57 years old. [^11^C]leucine scans (left column) represent cerebral tracer activity 30 to 60 min after injection. They show reduced temporal and parietal tracer uptake in the patient (bottom left). Scans do not show a quantitative image of rCPS but represent metabolized as well as unmetabolized tracer. Amyloid scans (right column) show non-specific tracer uptake in white matter only in the control subject (top right) but widespread cortical binding in the patient (bottom right). The color scale on the left represents PET tracer activity (arbitrary units).

Visual interpretation of [^11^C]leucine parametric images in individual AD patients also indicated that there might be a reduction of rCPS especially in parietal cortex. This was seen most clearly on static uptake [^11^C]leucine scans recorded 30 to 60 min after tracer injection (Fig. 5). AD patients typically also showed some degree of cortical atrophy, which might have caused partial volume effects on PET scans. This effect is likely very small, as we were using a dedicated high-resolution scanner with 2.5 mm intrinsic spatial resolution [49]. Performing accurate correction for partial volume effects is very difficult and controversial for dynamic PET studies on high resolution scanners [50], and we therefore did not perform corrections for potential partial volume effects.

Previous human PET studies aiming at rCPS using [^11^C]methionine did not find differences between AD patients and healthy controls [36] or a reduction in the mesial parietal cortex only [35]. However, [^11^C]methionine also enters other metabolic pathways and therefore may have lacked sensitivity to detect changes in rCPS [51]. In the TgF344-AD rat model of AD a reduction of rCPS was found with [11C]leucine PET primarily in the globus pallidus with a tendency towards reduction also in cortical areas [37].

The small number of subjects reported in this study is a major limitation. A larger study had been planned but could not be completed because of study interruption by the COVID-19 pandemic and a lack of production capacity for [^11^C]leucine at the University of Manchester’s Wolfson Molecular Imaging Centre after the COVID-19 pandemic.

In conclusion, this is the first study to demonstrate the feasibility of measuring protein synthesis dysfunction in Alzheimer’s disease using [^11^C]leucine PET. Further cross-sectional and longitudinal work is required to determine whether rCPS can reliably differentiate between Alzheimer’s disease and healthy ageing and thus ultimately provide a measure of efficacy in future clinical trials of disease modifying therapies. Our study provides guidance for planning of such studies, which should consider the data variance in older subjects and age-matched controls.

## Supplementary Information

Below is the link to the electronic supplementary material.

Supplementary file1 (DOCX 279 KB)
