# Supplementary material for: Quantitative Imaging of Regional Cerebral Protein Synthesis in Clinical Alzheimer's Disease by [11C]Leucine PET
Source: Mol Imaging Biol. 2024 Nov 20;26(6):977–85. doi: 10.1007/s11307-024-01965-3 (PMC11634943; doi:10.1007/s11307-024-01965-3)
Supplement: Supplementary file 1 — Supplementary file1 (DOCX 279 KB) [file 11307_2024_1965_MOESM1_ESM.docx]

**Supplementary materials**

**Quantitative Imaging of Regional Cerebral Protein Synthesis in Clinical Alzheimer's Disease by [^11^C]Leucine PET**

Karl Herholz, Adam McMahon, Jennifer C. Thompson, Matthew Jones, Herve Boutin, Jamil Gregory, Christine A. Parker, Rainer Hinz

# Methods (expanded)

## L-[1-^11^C]leucine production and quality control

L-[1-^11^C]leucine (termed [^11^C]leucine throughout the rest of the manuscript for brevity) was synthesized using a modified Bucherer-Strecker AA synthesis by the reaction of [^11^C]HCN on an aminosulfite derivative of isovaleradehyde to produce a racemic mixture of [^11^C]leucine enantiomers, based on the synthesis reported by Mu *et al*. [1]. Separation of the product [^11^C]leucine was performed by chiral HPLC (Chirobiotic T column (10 × 250 mm), ethanol/water: 5/95 (v/v), 3 mL/min) to give an enantiomeric purity > 96%. The production was carried out in a hot cell on a GE Tracerlab. [^11^C]HCN was produced by reaction of [^11^C]CH_4_ with ammonia on a platinum catalyst at a temperature between 900 and 950°C. [^11^C]methane was produced by reduction of the cyclotron produced [^11^C]CO_2_ with hydrogen over nickel catalyst at 400°C. [^11^C]methane and [^11^C]HCN were produced on a GE modules enclosed in a shielded cabinet. The decay-corrected radiochemical yield was 23.8 ± 6.8% on the basis of trapped [^11^C]HCN in the reaction vial.

## PET scanning ([^11^C]leucine)

All PET scans were performed at the Wolfson Molecular Imaging Centre (WMIC) on a High Resolution Research Tomograph (HRRT, CTI/Siemens Molecular Imaging, Knoxville, Tennessee), a dedicated 3D human brain camera with an axial field of view of 25 cm and 207 reconstructed transaxial image planes [2]. A 7 min transmission scan using a ^137^Cs point source was acquired for subsequent attenuation and scatter correction. After the start of the emission scan acquired in list mode, [^11^C]leucine was injected intravenously as a slow bolus of 10 ml over approximately 30 s followed by a flush of heparinised saline. The injected radioactivity dose was between 520 and 783 MBq (mean: 641 MBq, SD: 66 MBq). The radiochemical purity of the injected [^11^C]leucine was high and ranged from 98.0 % to 99.4 % with a mean of 98.8 % and a SD of 0.6 %. The injected mass of leucine varied between 2.6 and 19.8 μg with a mean value of 7.4 μg and a SD of 5.4 μg. The specific activity was on average 16.4 GBq · μmol^-1^ with a SD of 8.6 GBq · μmol^-1^.

The 90 min emission scan was binned into 43 frames: a background frame of approx. 7 min before the injection, then 16 frames of 15 s, 4 frames of 30 s, 4 frames of 60 s, 4 frames of 150 s and 14 frames of 300 s [3]. The head position of the participant in the PET camera was indicated using laser beams and monitored via an optical camera throughout the scanning session and corrected immediately if necessary.

The dynamic PET images were reconstructed with the iterative ordinary Poisson ordered-subset expectation maximization (OP OSEM) 3-D algorithm, incorporating normalization and corrections for random coincidences, scattered radiation and attenuation [4]. 16 subsets and 12 iterations were used for the mean activity concentration at the volume of interest level to converge. The voxel size of the reconstructed PET images was 1.22 × 1.22 × 1.22 mm^3^. After reconstruction, images were regularized with a 3-D Gaussian filter of 2 mm full-width at half-maximum to reduce image noise.

Arterial whole-blood activity was monitored continuously for the first 15 min of the scan with a bismuth germanate coincidence detector [5]. A total of 11 discrete arterial blood samples were taken at 5, 10, 15, 20, 30, 40, 50, 60, 70, 80 and 90 mins into heparinised syringes. The activity concentration of the whole blood and plasma were measured.

Nine plasma samples per scan (at 5, 15, 30, 40, 50, 60, 70, 80 and 90 min) were analysed for the parent fraction of [^11^C]leucine unbound to plasma protein. Free [^11^C]leucine in plasma was determined using an adaptation of the method reported by Keen et al. [6]. Plasma proteins were precipitated by the addition of 5% perchloric acid (1:1 v/v) and samples were gently swirled for 30s. Samples were then centrifuged at 2000g for 3 min and the pellet separated from the supernatant. Activity in the supernatant (free [^11^C]leucine) was measured in a NaI well counter. [^11^C] CO_2_ was not measured, as the concentration in plasma samples are known to be negligible [7].

## Generation of plasma input functions

For the generation of the plasma input functions, the time course of the plasma to whole blood activity ratio, obtained from the 11 discrete arterial samples, was first fitted to the model function [8]

$$y\left( t \right)={(1+\left( x_{1}t \right)^{2})}^{{-x}_{2}}-x_{3}$$

with *x*_1_ > 0, *x*_2_ < 0 and *x*_3_ < 0 for a monotonically increasing plasma-over-blood ratio.

Then the measurement of the arterial whole blood activity obtained from the continuous detector system [5] was multiplied with the function values *y* (*t*) for 0 < *t* < 15 min to obtain a total plasma activity curve for the first 15 min of the scan. This curve was then combined with a spline interpolation of the discrete plasma activity concentration measurements to generate an input function describing the total plasma activity concentration for the entire scan.

The input function of the activity concentration due to unbound [^11^C]leucine in plasma was then created by multiplying the total plasma activity input function with the function describing the fraction of unbound [^11^C]leucine in plasma with time.

A population mean curve for the fraction of unbound [^11^C]leucine in plasma as published by Sundaram *et al.* [9] was used to convert the total plasma activity input function into the parent in plasma activity input function.

$$y\left( t \right)=offset+ \frac{1-offset}{1+e^{-\alpha+\beta t}}$$

with *offset*= 0.132, *α*= 2.861 and *β*= 0.0016 [9].

Finally, the time delay of the arrival of the radioactivity bolus at the peripheral sampling site relative to the brain was determined [10]. All calculations were performed using in-house software based on Matlab® (The MathWorks, Inc., Natick, MA, USA).

## Magnetic Resonance Scans and Definition of Volumes of Interest

For each participant, brain magnetic resonance images (MRI) were acquired to provide structural volumetric T1 images for subsequent segmentation and atlas-based generation of volumes of interest. MP-RAGE (Magnetization Prepared - RApid Gradient Echo) images in sagittal orientation were acquired on a 1.5 Tesla Philips Achieva scanner. The T1 images in sagittal orientation had square pixels of (0.9375 mm)^2^ with 1.2 mm slice distance (140 planes with 256 × 256 pixels). To rule out any gross abnormalities of the brain, additional T2 weighted images were acquired and reviewed.

A set of volumes of interest (VOI) was defined with a probabilistic brain atlas template [11]. Fourteen bilateral VOIs adapted from the report by Bishu *et al.* [12] were chosen for the kinetic analysis of [^11^C]leucine:

- Nine grey matter VOIs (cerebellum, occipital cortex, prefrontal cortex, orbitofrontal cortex, parietal cortex, temporal cortex, precentral gyrus, postcentral gyrus, anterior cingulate),
- Four unsegmented tissue VOIs (hippocampus, amygdala, thalamus, putamen),
- One white matter VOI (corpus callosum).

After co-registering the individual MRI to the PET image summed from 30 to 60 min after tracer injection [12] using the linear image registration tool [13], regional time–activity curves were generated from the dynamic images using the medical imaging software ANALYZE [14].

## Quantification of [^11^C]leucine in the brain

The approach used for the quantification of [^11^C]leucine in brain tissue was based on the previous work by Hawkins *et al.* [7], Sundaram *et al.* [9] and Bishu *et al.* [12]. An irreversible two-tissue compartment model with three rate constants as specified by Sundaram *et al.* [9] and the fractional blood volume as free model parameter was used for the calculation of the following outcome parameters:

1. Unidirectional uptake rate constant of plasma leucine into tissue

*K*_cplx_= *K*_1_ *k*_3_ / (*k*_2_ + *k*_3_)

1. Fraction of intracellular leucine originating from plasma

*λ* = *k*_2_ / (*k*_2_ + *k*_3_)

1. Normalised unidirectional uptake rate constant

$$K_{cplx}^{'}=K_{cplx}\frac{LNAA (\mu M)}{1000 \mu M}$$

2. Protein synthesis rate (rCPS)

$$rCPS\left( \mu M{\cdot min}^{-1} \right)=K_{cplx}({min}^{-1})\frac{leucine(\mu M)}{\lambda}$$

When using K’_cplx_ in this equation, rCPS normalised to a standard total sum of all large neutral amino acids (LNAAs ) concentration is obtained, denoted PSR′ by Sundaram *et al.* [9] and rCPS (normalised) in the present report.

LNAA denotes the sum of the plasma concentrations of these nine large neutral AA: leucine, threonine, glutamine, tyrosine, histidine, valine, phenylalanine, methionine and isoleucine [9]. Two plasma samples were taken from each participant (one sample after the insertion of the venous access cannula in preparation of the PET scan, the second sample upon completion of the PET scanning procedure) and then sent for free AA analysis by high resolution chromatography to Alta Bioscience (Redditch, UK).

Estimates of compartmental model parameters were obtained from weighted fits of the regional tissue time-activity curves as previously described [8]. The calculations were performed with in house software based on Matlab (The MathWorks, Inc., Natick, Massachusetts, USA). Non-linear least squares estimation was performed with the data uncorrected for radioactive decay using the weighting scheme [18] and the Levenberg-Marquardt algorithm [19] until the termination tolerance was reached. All fits of regional time-activity curves were visually inspected.

As this study adopted the methodology used by Sundaram *et al*. [9] in healthy adults aged 20 to 50 years, only the irreversible two-tissue compartment model with three rate constants and the fractional blood volume as free model parameter was used. A formal comparison between the performance of different compartmental model configurations was published by Hawkins *et al.* [7] and subsequently extended through the use of spectral analysis by Veronese *et al.* [20].

## PET scanning ([^18^F]flutemetamol)

Older healthy subjects and AD patients also underwent an amyloid PET scan with 150 MBq [^18^F]flutemetamol (GE Healthcare, Amersham, UK) via a slow bolus intravenous injection. A PET scan of the cerebral deposition of beta amyloid was then recorded 90 to 110 minutes after tracer injection. Scans were evaluated visually for pathological deposition of amyloid according to standard clinical criteria [15].

# Additional results

## Plasma amino acids

Analysis of unlabelled AAs showed results similar to previous reports in the literature [16-17] (Tables 2a and 2b). There was a tendency towards higher values in male than in female participants, and young healthy subjects tended to have lower levels than older healthy subjects, but none of these differences reached significance. Levels were measured twice in each subject, before and after the PET scan. There was a tendency of LNAA levels having higher values at the end of the scan compared to before the scan (LNAA levels: 1188.79 ± 125.11 µmol/L prior to scan; and 1255.96 ± 145.01 µmol/L after scan; mean ± SD; *p*=0.096 in paired *t*-test), which reached significance for leucine (104.92 ± 13.61 µmol/L prior to scan vs 115.47 ± 16.61 µmol/L after scan, *p*=0.048). Averages of the two measurements were used for calculation of rCPS.

## Radiotracer and metabolites in plasma

An initial analysis of measurements of protein-bound and parent fraction ^11^C activity in plasma samples showed significant differences from data published by Sundaram et al. [9]. While we typically found still about 50% of [^11^C]leucine activity in the plasma parent fraction after 60 minutes, the data presented by Sundaram et al. [9] demonstrated the parent fraction to be at 20% at the same time-point. This resulted in substantially lower rates of rCPS and substantially higher estimates of the AA fraction in the intracellular precursor pool (λ) in our controls than in the literature reports [9, 12]. Experimental variation of the method used for protein precipitation in subsequent scans with variation of the relative volumes and concentration of perchloric acid had a substantial effect on the measurement of the parent fraction, indicating a high sensitivity and variation of results depending on technical parameters. We therefore decided to abandon the individual measurements of the parent fraction and use the published population time course [9] throughout for data analysis.

# Supplementary references

1. Mu F, Mangner TJ, Chugani HT (2005) Facile Synthesis of L-[1-C-11] Leucine as a PET Radiotracer for the Measurment of Cerebral Protein Synthesis. Journal of Labelled Compounds and Radiopharmaceuticals pp S189-S189.

2. de Jong HW, van Velden FH, Kloet RW, Buijs FL, Boellaard R, Lammertsma AA (2007) Performance evaluation of the ECAT HRRT: an LSO-LYSO double layer high resolution, high sensitivity scanner. Phys Med Biol 52:1505-1526.

3. Tomasi G, Bertoldo A, Bishu S, Unterman A, Smith CB, Schmidt KC (2009) Voxel-based estimation of kinetic model parameters of the L-[1-(11)C]leucine PET method for determination of regional rates of cerebral protein synthesis: validation and comparison with region-of-interest-based methods. J Cereb Blood Flow Metab 29:1317-1331.

4. Hong IK, Chung ST, Kim HK, Kim YB, Son YD, Cho ZH (2007) Ultra fast symmetry and SIMD-based projection-backprojection (SSP) algorithm for 3-D PET image reconstruction. IEEE transactions on medical imaging 26:789-803.

5. Ranicar AS, Williams CW, Schnorr L, et al. (1991) The on-line monitoring of continuously withdrawn arterial blood during PET studies using a single BGO/photomultiplier assembly and non-stick tubing. Medical Progress through Technology 17:259-264.

6. Keen RE, Barrio JR, Huang SC, Hawkins RA, Phelps ME (1989) In vivo cerebral protein synthesis rates with leucyl-transfer RNA used as a precursor pool: determination of biochemical parameters to structure tracer kinetic models for positron emission tomography. Journal of Cerebral Blood Flow & Metabolism 9:429-445.

7. Hawkins RA, Huang SC, Barrio JR, et al. (1989) Estimation of local cerebral protein synthesis rates with L-[1- 11C]leucine and PET: methods, model, and results in animals and humans. Journal of Cerebral Blood Flow & Metabolism 9:446-460.

8. Hinz R, Bhagwagar Z, Cowen PJ, Cunningham VJ, Grasby PM (2007) Validation of a tracer kinetic model for the quantification of 5-HT(2A) receptors in human brain with [(11)C]MDL 100,907. J Cereb Blood Flow Metab 27:161-172.

9. Sundaram SK, Muzik O, Chugani DC, Mu F, Mangner TJ, Chugani HT (2006) Quantification of Protein Synthesis in the Human Brain Using L-[1-11C]-Leucine PET: Incorporation of Factors for Large Neutral Amino Acids in Plasma and for Amino Acids Recycled from Tissue. J Nucl Med 47:1787-1795.

10. Hinz R, Turkheimer FE (2006) Determination of tracer arrival delay with spectral analysis. IEEE transactions on nuclear science 53:212-219.

11. Hammers A, Allom R, Koepp MJ, et al. (2003) Three-dimensional maximum probability atlas of the human brain, with particular reference to the temporal lobe. Human Brain Mapping 19:224 - 247.

12. Bishu S, Schmidt KC, Burlin T, et al. (2008) Regional rates of cerebral protein synthesis measured with L-[1-11C]leucine and PET in conscious, young adult men: normal values, variability, and reproducibility. J Cereb Blood Flow Metab 28:1502-1513.

13. Jenkinson M, Bannister P, Brady M, Smith S (2002) Improved optimization for the robust and accurate linear registration and motion correction of brain images. Neuroimage 17:825-841.

14. Robb RA, Barillot C (1989) Interactive display and analysis of 3-D medical images. IEEE Trans Med Imaging 8:217-226.

15. Duara R, Loewenstein DA, Shen Q, et al. (2013) Amyloid positron emission tomography with 18F-flutemetamol and structural magnetic resonance imaging in the classification of mild cognitive impairment and Alzheimer’s disease. Alzheimer's & dementia : the journal of the Alzheimer's Association 9:295-301.

16. Caballero B, Gleason RE, Wurtman RJ (1991) Plasma amino acid concentrations in healthy elderly men and women. Am J Clin Nutr 53:1249-1252.

17. Basun H, Forssell LG, Almkvist O, et al. (1990) Amino acid concentrations in cerebrospinal fluid and plasma in Alzheimer's disease and healthy control subjects. Journal of Neural Transmission-Parkinson's Disease and Dementia Section 2:295-304.

18. Gunn RN, Sargent PA, Bench CJ, Rabiner EA, Osman S, Pike VW, Hume SP, Grasby PM, Lammertsma AA (1998) Tracer kinetic modeling of the 5-HT1A receptor ligand [carbonyl-11C]WAY-100635 for PET. Neuroimage 8:426–40.

19. Marquardt DW (1963) An algorithm for least-squares estimation of nonlinear parameters. J Soc Ind Appl Math 11: 431-441.

20. Veronese M, Bertoldo A, Bishu S, Unterman A, Tomasi G, Smith CB, Schmidt KC (2010) A spectral analysis approach for determination of regional rates of cerebral protein synthesis with the L-[1-^11^C]leucine PET method. J Cereb Blood Flow Metab 30: 1460 - 1476.

# Supplementary figure legends

Figure 1: Example time course of [11C]leucine plasma free fraction measured in plasma samples from a young volunteer in the present study (red dots) compared to a population curve (green) published by Sundaram et al. [9].

Figure 2: Proportion of calculated intracellular fraction of plasma-derived leucine, λ (box plot)

# Supplementary figures

**
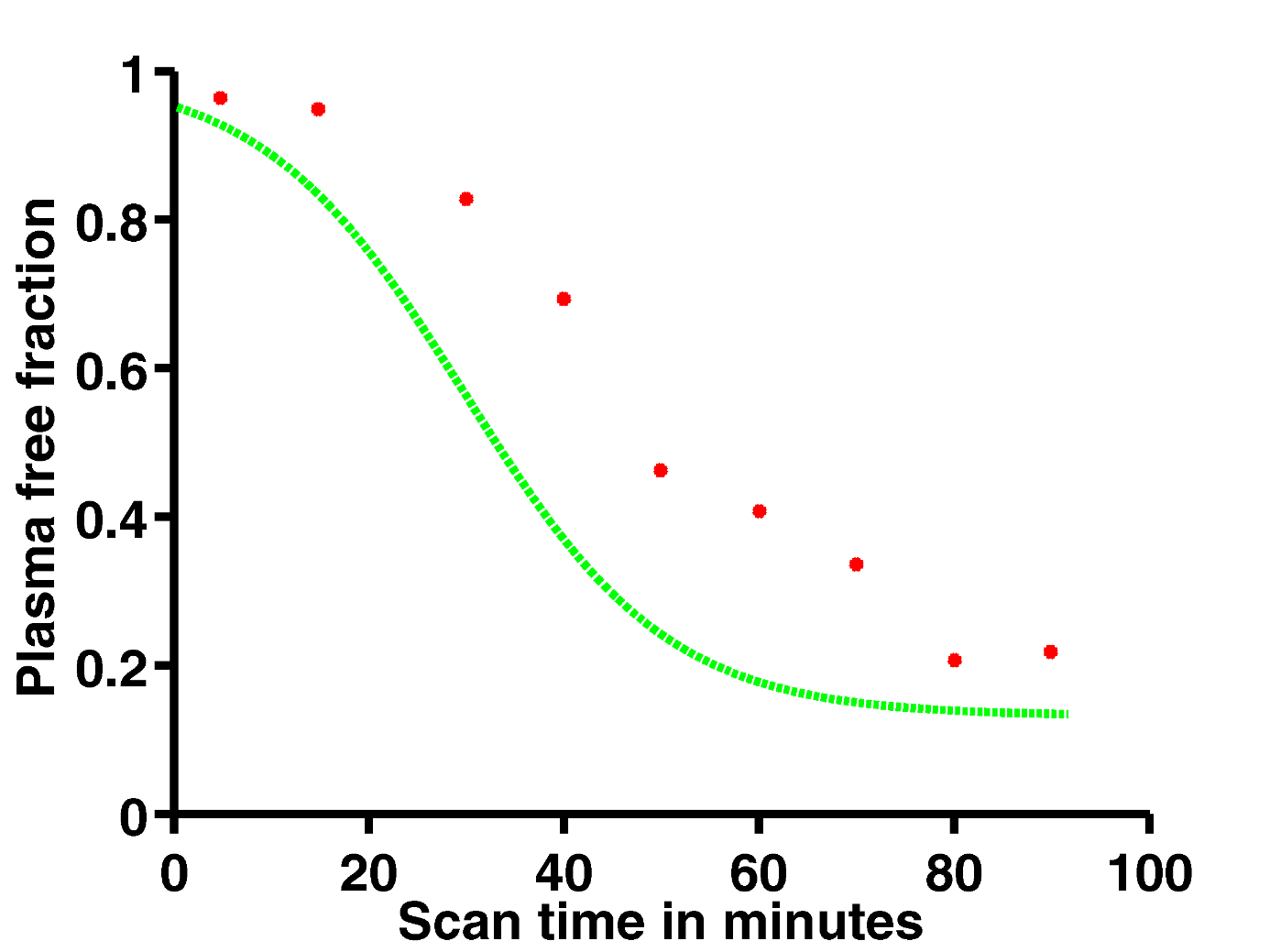
**

Figure 1: Example time course of [^11^C]leucine plasma free fraction measured in plasma samples from a young volunteer in the present study (red dots) compared to a population curve (green) published by Sundaram et al. (23).


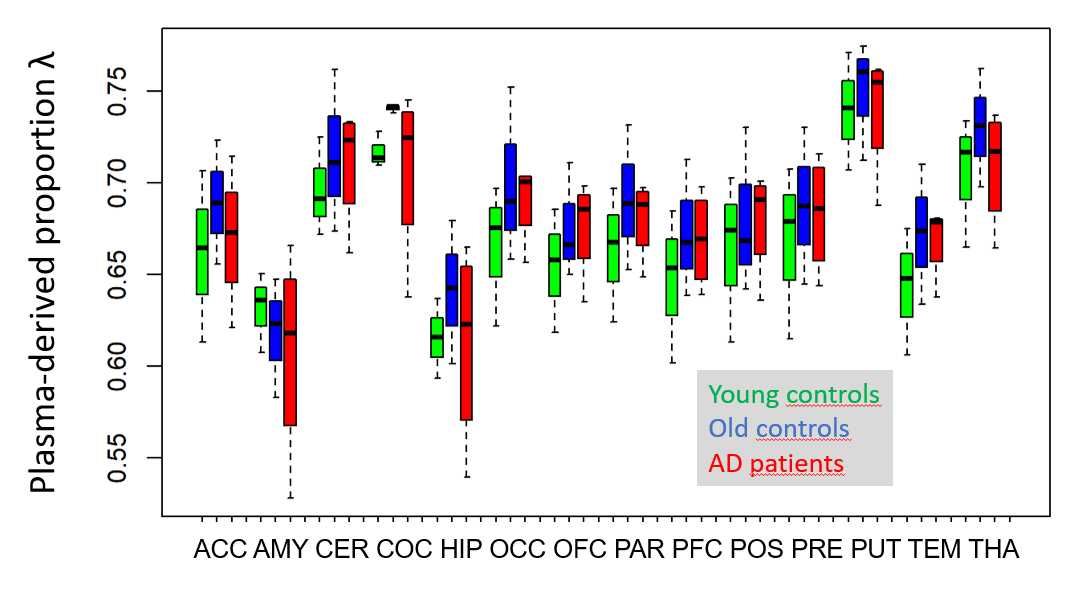


Figure 2: Proportion of calculated intracellular fraction of plasma-derived leucine (box plot)
